# The Effect of a Happiness Training Program on Sense of Coherence and Perceived Fatigue in Patients With Type II Diabetes

**DOI:** 10.1002/hsr2.72198

**Published:** 2026-04-28

**Authors:** Mohammad Hassan Ahmadi Sartakhti, Mahdieh Poodineh Moghadam, Najmeh Ahmadpour, Tayebe Sargazishad

**Affiliations:** ^1^ Students Research Committee, Faculty of Nursing and Midwifery Ilam University of Medical sciences Ilam Iran; ^2^ Department of Nursing, Faculty of Nursing and Midwifery Zabol University of Medical Sciences Zabol Iran; ^3^ Students Research Committee, Faculty of Nursing and Midwifery Zabol University of Medical sciences Zabol Iran

**Keywords:** happiness training, perceived fatigue, sense of coherence, type II diabetes

## Abstract

**Background and Aims:**

Patients with type II diabetes often face numerous challenges, including fatigue and decreased sense of coherence (SOC). Given the chronic nature of this disease, changing the mental health aspects can be one of the most significant changes for individuals with type II diabetes. Therefore, this research aimed to study the effect of a happiness training program on the SOC and perceived fatigue among individuals with type II diabetes.

**Methods:**

This semi‐experimental research involved two groups and was performed on 72 participants diagnosed with type II diabetes. Eligible patients were recruited through a convenience sampling method. Subsequently, participants were randomly allocated to either the intervention or control group using a lottery‐based method, ensuring equal probability of assignment for each individual. This randomization helped reduce selection bias and ensure comparable groups. After obtaining informed consent, the demographic questionnaire, the multidimensional fatigue inventory (MFI), and Antonovsky's SOC scale were completed. The intervention group received happiness skills training intervention in a group for 10 1‐h sessions, twice a week, and the control group received routine interventions of the center. Data analysis was performed in SPSS software.

**Results:**

The mean SOC value significantly improved in the intervention group from 53.81 ± 9.74 to 65 ± 8.48, which was significant as indicated by the paired *t*‐test (*p* = 0.000). Moreover, the mean perceived fatigue score in the intervention group reached from 61.32 ± 15.55 to 53.67 ± 15.29, which was statistically significant, as indicated by the results of the paired *t*‐test (*p* = 0.000).

**Conclusions:**

It can be inferred that happiness training contributes to enhancing the SOC and reducing perceived fatigue to enhance and cultivate a healthy relationship with the self, healthy interpersonal relationships in life, and self‐confidence.

## 1 Introduction

Type II diabetes is a chronic, multifactorial disorder characterized by hyperglycemia and long‐term damage to organs and tissues [1]. It arises from a state of insulin resistance combined with relative insulin deficiency [2], and its classic symptoms include polydipsia, polyuria, and polyphagia [3].

As stated by the World Health Organization (WHO), the number of adults aged 20 and older with diabetes is expected to reach 300 million by 2025 [4]. Diabetes has a highly variable geographical distribution worldwide [5]. Currently, the number of diabetic individuals in Iran is estimated at 1.5 million [6]. Recent studies conducted by the WHO indicate that in Iran, diabetes and its associated risk factors affect 11.1% of women, 9.6% of men, and 10.3% of the population overall [7]. Epidemiological studies have shown that the distribution of diabetes in Iran is variable [8].

This chronic disease affects an individual's physical and mental health, as well as their overall functioning; therefore, its investigation is of particular importance [9]. Patients with type II diabetes experience numerous problems, one of which is fatigue. Fatigue is an undefined feeling and a subjective complaint of weariness that differs from feeling a lack of energy due to apathy or sleepiness [10]. Findings of a study performed by Bayat Asghari et al. revealed that fatigue, with its complex and profound effects on the psychological well‐being of individuals with chronic illnesses, leads to depression and decreased life expectancy [11].

In fact, psychological tensions can significantly contribute to increasing blood sugar levels, and also diabetes, from various aspects, such as increased medical care costs, increased hospital admissions, hypoglycemia, and increased ketoacidosis, culminate in increased psychological problems and decreased levels of happiness among patients [11, 12].

In the science of psychology, terms like “psychological well‐being” and “sense of coherence” are considered key components of the psychology of quality of life. Psychological well‐being refers to how an individual perceives their life in relation to their emotional behaviors and psychological functioning related to mental health dimensions. Happiness, on the other hand, is a pleasant and delightful personal state arising from the experience of positive emotions; thus, happiness is the product of individuals' positive judgments about their lives, judgments that are not imposed from the outside but are rather internal states [13].

According to Antonovsky, three components constitute the sense of coherence (SOC): (1) Comprehensibility, refers to the development of one's understanding that the world is predictable and comprehensible; (2) manageability, defined as the development of an individual's perception of having access to coping resources and the ability to manipulate realities; and (3) meaningfulness, defined as the development of an individual finding emotional meaning, challenges, and interests in their life [14].

An important issue in the lives of diabetic individuals is a change in happiness and mental health. Positive psychology has opened up new horizons for psychologists and researchers. One of the core concepts in positive psychology, which is itself a relatively new field of psychology, is happiness [15].

Happiness can act as a shield, protecting individuals from stress. Moreover, happiness generates energy, enthusiasm, and emotion, and ensures individuals' well‐being [16].

Results of the research performed by pelechano et al. revealed that fatigue, with its complex and profound effects on the psychological well‐being of individuals with chronic illnesses, leads to depression and decreased life expectancy [17].

Due to the scarcity of studies investigating the effects of happiness training on the SOC and perceived fatigue in patients with type II diabetes, recommendations for further research in this area, and also the positive effects of happiness on life expectancy and quality of life, researchers were motivated to conduct a study aimed at determining the effects of happiness training on the SOC and perceived fatigue among type II diabetes patients in Zabol, Iran, from 2023 to 2024.

## 2 Methods

This semi‐experimental research was conducted on two groups using a pretest‐posttest approach. The study population included type II diabetes patients. The study sample comprised type II diabetes patients with an active medical record at the Zabol Diabetes Clinic during 2023–2024. After obtaining the necessary permits and approval from the ethics committee, the research team proceeded to collect the required data from the research units. The sampling was performed using the convenience sampling method. Subsequently, patients were allocated to the intervention and control groups through simple random sampling and lottery methods. The sample size was estimated according to an independent‐samples *t*‐test (two‐tailed) for comparing two groups using G*Power software, assuming a medium effect size (Cohen's *d* = 0.5), a power of 0.80, and a significance level of 0.05. A sample size of 36 participants per group was determined.

The inclusion criteria included type II diabetes, at least a basic level of literacy, signed informed consent to participate in the study, and willingness to learn happiness skills. The exclusion criteria included no mental disorders (such as acute depression or generalized anxiety disorder), no other chronic diseases (such as cancer), no previous participation in similar training programs, unwillingness to continue participating in the study, and participation in similar training programs within the past 6 months. The ineligible participants were excluded at the beginning of the research.

Before data collection, participants were provided with detailed explanations regarding the study's aims and how to complete the questionnaires. After written informed consent was obtained, both groups completed the multidimensional fatigue inventory (MFI) and Antonovsky's SOC scale. Happiness skills group training intervention was provided for the intervention group in 10 1‐h sessions, twice a week, based on previous studies (Table 1) [18].

**Table 1:** A summary of the happiness skills training program for patients with type II diabetes.

Session	Treatment content
One	Administering pretest, the MFI, and the SOC scale, along with a brief explanation of group therapy, its objectives, and the upcoming procedures
Two	Introducing oneself and familiarizing with others, familiarizing session participants with each other, introducing the procedure and the number of sessions, and training and implementing goal‐setting technique
Three	Introducing the happiness formula, the overall introduction of happiness techniques, and presenting a technique to increase physical activity
Four	Providing the happiness formula and offering techniques to enhance social skills
Five	Training in emotion expression techniques, cultivating optimism and positive thinking, reducing worry‐related thoughts, and emphasizing the importance of happiness
Six	Social problem‐solving abilities and creativity in building a relationship and clarifying it using examples, providing techniques to enhance social problem‐solving abilities and creativity in building relationships with oneself and society
Seven	Escaping from worry, training techniques to reduce expectations techniques to gratitude, both verbally and through actions
Eight	Training techniques to increase social intimacy and providing techniques for concentrating on the present moment and living in the moment
Nine	Focusing on enhancing physical activity, teaching planning strategies, offering techniques for prioritizing happiness, and reviewing
Ten	Administering posttest and gratitude to the participants in the design.

Abbreviations: MFI, multidimensional fatigue inventory; SOC, sense of coherence scale.

The effect of this training on the SOC and perceived fatigue was reassessed at the beginning and the end of the sessions. The control group underwent the routine interventions of the center. After the specified timeframe, both groups re‐completed the questionnaires.

The tools utilized in this study encompassed a patient demographic and clinical data questionnaire, the MFI, and the SOC scale.

The MFI, established by Smets et al., was employed to evaluate fatigue. This scale is recognized as one of the most thorough and comprehensive instruments for the assessment of fatigue [19], which offers enhanced depth and precision in the understanding of an individual's fatigue levels by evaluating five aspects of fatigue: physical fatigue, general fatigue, mental fatigue, reduced motivation, and reduced activity. In essence, the MFI measures fatigue as it is perceived and reported by the individual [20].

This questionnaire includes 20 items that are scored according to a 5‐point Likert scale (ranging from “1 = completely true” to “5 = completely false”). It should be mentioned that the higher overall scores reflect higher levels of fatigue [20].

Validation of the Persian version of this questionnaire has been demonstrated in research performed by Najafi Mehri et al. and Hafezi et al. reported a Cronbach's alpha of 0.85 for the MFI [20, 21]. Zadi Akhule and Nasiri used Cronbach's alpha to evaluate the reliability of this scale, which was calculated at 0.87 [22].

SOC scale consists of a 13‐item form (SOC‐13). The items were scored according to a 7‐point Likert scale ranging from 1 to 7. The total score represents the individual's overall score on the test [11].

In a systematic review, Erikson and Lindström concluded that the SOC (SOC‐13) scale is reliable and valid and has cross‐cultural applicability. The alpha coefficients obtained for the scale across 124 studies were within the range of 0.70–0.95. Test‐retest reliability indicated the stability of the scale with correlations of 0.69–0.78 over 1 year, 0.64 over 2 years, 0.42–0.45 over 4 years, 0.59–0.67 over 5 years, and 0.54 over 10 years [23]. Studies carried out in Iran have confirmed the reliability and validity of this tool. Alipour and Sharif adapted and standardized the questionnaire, reporting Cronbach's alpha coefficients of 0.75 and 0.78. The test‐retest reliability coefficient for the total scale was 0.66. Furthermore, to assess the questionnaire's validity, these researchers examined correlations between the subscales and the total scale, obtaining coefficients of 0.86 and 0.87, respectively, indicating the scale's satisfactory validity [24].

The Shapiro–Wilk test was employed to evaluate the normality of continuous variables. Normally distributed variables between the two groups were compared using the independent samples *t*‐test. Moreover, a nonparametric Kruskal–Wallis test was used for variables that did not meet the normality assumption or for comparisons involving more than two groups. Statistical significance was set at a *p*‐value below 0.05.

After collecting the relevant data and entering them into SPSS Version 23, analysis was performed using descriptive statistics (mean, standard deviation, and relative frequency distribution) and inferential statistics. In this research, statistical significance was defined as *p* < 0.05, and a 95% confidence interval was considered.

### 2.1 Ethics Approval

All ethical requirements, such as obtaining written informed consent from participants, assuring them of the confidentiality of their personal data, and anonymous use of their data, were strictly adhered to throughout the study. Throughout the entire process, principles of confidentiality, privacy, and respect were upheld according to the Declaration of Helsinki. Necessary permissions were obtained, and approval was granted by the Ethics Committee of Zabol University of Medical Sciences (IR.ZBMU.REC.1402.111) to conduct this research.

This research was not registered in a clinical trial registry before its implementation. At the time of study initiation, the research team was not fully aware of the registration requirements for behavioral or nonpharmacological interventions, especially those conducted as part of academic or student theses. Additionally, the study did not involve any drug, device, or invasive procedure, and was conducted with minimal risk to participants. However, we acknowledge the importance of trial registration for transparency and research integrity, and we will ensure compliance with this practice in future studies.

## 3 Results

All patients were included in the final analysis, and none were excluded from the research. The present research included 24 women (64.9%) and 13 men (35.1%) with type II diabetes in the combined intervention and control groups. The rest of the demographic information, including the genders, age, occupation, the marital status and literacy levels are presented in the Table 2.

**Table 2:** Demographic characteristics of participants in the intervention and control groups.

Variable	Group	Frequency (%)/Mean ± SD	Statistical test	*p*‐value
Gender	Intervention	Female: 24 (64.9%) Male: 13 (35.1%)	Chi‐square test	0.07
	Control	Female: 24 (64.9%) Male: 13 (35.1%)		
Age (years)	Intervention	55.97 ± 8.32	Independent *t*‐test	0.715
	Control	56.65 ± 9.98		
Marital status	Intervention	Married: 35 (94.6%)	Kruskal–Wallis test	0.04
	Control	Married: 34 (91.9%)		
Education level	Intervention	Elementary: 20 (54.1%)	Kruskal–Wallis test	0.480
	Control	Elementary: 17 (45.9%)		

As shown in the table, the Kruskal–Wallis test indicated no statistically significant differences between the intervention and control groups regarding occupation (*p* = 0.489).

The patients had a mean diabetes duration of 11.91 ± 7.72 years in the intervention group and 9.86 ± 6.57 years in the control group. According to the independent *t*‐test, the mean scores were not significantly different between the two groups, and the participants were homogeneous regarding age (*p* = 0.222).

Most of the patients were complication‐free, including 22 (59.5%) and 16 (43.2%) individuals in the intervention and control groups, respectively. The Kruskal‐Wallis test revealed the homogeneity of samples regarding physical complications between the intervention and control groups (*p* = 0.224).

Also, the majority of participants had a moderate socioeconomic status, with 18 (48.6%) and 23 (62.2%) individuals in the intervention and control groups, respectively. Moreover, the Kruskal‐Wallis test showed no statistically significant differences between the socioeconomic status of the two groups (*p* = 0.880), which means that the samples in both groups were homogeneous regarding socioeconomic status.

The mean fasting blood glucose levels of type II diabetes patients in the intervention and control groups were 125.12 ± 249.83 and 77.43 ± 221.78 mg/dL, respectively. Mann‐Whitney *U*‐test revealed no significant difference in mean blood glucose levels between the intervention and control groups, indicating sample homogeneity (*p* = 0.677).

The mean values of the SOC score at the baseline were 53.81 ± 9.74 and 50.43 ± 13.45 for the intervention and control groups, respectively. According to the independent samples t‐test, there was no significant difference between the two groups at the baseline in the SOC (*p* = 0.220, df = 72). The mean SOC scores after the intervention were 65 ± 8.48 and 46.72 ± 13.38 in the intervention and control groups, respectively. According to the independent samples *t*‐test, there were significant changes in SOC postintervention (*p* = 0.000, df = 72).

The mean SOC score in the intervention group increased from 53.81 ± 9.74 to 65 ± 8.48. The paired *t*‐test indicated that this increase was significant (*p* = 0.000, df = 36). In addition, the mean SOC score reached from 50.43 ± 13.45 to 46.72 ± 13.38 in the control group, which was significant based on the paired *t*‐test (*p* = 0.003, df = 36).

The mean perceived fatigue scores before the intervention were 61.32 ± 15.55 and 58.37 ± 15.88 in intervention and control groups, respectively. Based on an independent *t*‐test, the perceived fatigue did not differ significantly between the two groups at the baseline (*p* = 0.423). The means of perceived fatigue scores after the intervention were 53.67 ± 15.29 and 43.59 ± 10.67 in the intervention and control groups, respectively. The independent samples *t*‐test showed significant differences in perceived fatigue postintervention (*p* = 0.05).

The mean perceived fatigue score in the intervention group reached from 61.32 ± 15.55 to 53.67 ± 15.29. The paired t‐test indicated that this change was significant (*p* = 0.000). The mean perceived fatigue score of the control group reached from 58.37 ± 15.88 to 59.43 ± 10.67; however, the paired *t*‐test revealed that this change was not significant (*p* = 0.698) (Table 3).

**Table 3:** Comparison of mean perceived fatigue scores between the intervention and control groups pre and postintervention.

		Mean (standard deviation)				
Perceived fatigue	Frequency	Intervention group	Control group	Independent *t*‐test	df	Significance level
Before intervention	37	61.32 ± 15.55	58.37 ± 15.88	0.806	72	0.423
After intervention	37	53.67 ± 15.29	59.43 ± 10.67	−1.80	72	0.05
		*p* = 0.000 df = 36 *t* = −0.391 paired *t*‐test	*p* = 0.698 df = 36 *t* = −5.44 paired *t*‐test			

## 4 Discussion

Determining the effects of happiness training on the SOC and perceived fatigue among type II diabetes patients, the findings of the present research revealed that happiness training improved the SOC among these patients.

This result aligns with those of the research conducted by Mikaeili and Rahimzadegan and Hosseini and Abdekhdaei, demonstrating the effectiveness of the happiness training program in increasing the SOC in diabetic patients [18, 25].

Furthermore, the present results align with those of Kaivand et al. study on diabetic patients, demonstrating the effectiveness of Fordyce's happiness training in increasing happiness [26].

Additionally, in a study on adolescents who survived cancer, Bitsko et al. indicated that happiness significantly influenced the adolescents' quality of life, depressive symptoms, and the severity of their treatment [27].

Observations of the present study support the results of Siomon et al. study, investigating the systematic treatment of depression in diabetic individuals and its reduced treatment costs [28].

They also align with the findings of Mirzaei Teshnizi et al. research that compared the effectiveness of training the subjective well‐being program and Fordyce's cognitive‐behavioral method in reducing depression and anxiety [29]. Furthermore, Pignone's research supports the present study results regarding the impact of happiness training on social functioning in individuals with diabetes [30].

The results of Rajabi and Abbasi's study demonstrated that cognitive‐behavioral training improved happiness, and Fordyce's cognitive‐behavioral method led to mitigating migraine headaches and promoting overall happiness in individuals; thus, such psychological interventions can be considered a complementary treatment to medication [31].

A group‐based approach to promoting happiness may enable individuals to better manage their diabetes through the use of specific techniques. What matters more than stress, anxiety, and depression in the diabetes process is the individual's response type to the factors causing these concerns regarding happiness training [32].

McCarty et al. also suggest in their study that psychological interventions that reduce negative reactions and enhance positive states significantly impact cardiovascular functioning, and positive emotions can be effective in treating hypertension, heart failure, and coronary artery disease [33].

As shown in Hurtado's study, a high SOC enhances the tendency to control stress better and more effectively, leading the individual to feel higher levels of well‐being. Conversely, a low SOC increases vulnerability to illness [34].

Another finding of this study was that happiness program training was effective in reducing perceived fatigue in patients with type II diabetes. This result aligns with the findings of studies conducted by Mikaeili and Rahimzadegan and Konen. Konen found that a group‐based happiness increase approach helped individuals manage their diabetes using specific techniques. Mikaeili and Rahimzadegan also concluded that Fordyce's happiness training program was effective in reducing perceived fatigue in diabetic patients [18, 32].

Training patients to observe disturbing thoughts and emotions without judgment, to reframe them in a more positive light, and to accept rather than avoid or ruminate on them has culminated in enhanced self‐awareness, increased happiness, the development of more conscious and adaptive responses, and improved management of unpleasant thoughts and emotions in diabetic patients. Ongoing cognitive‐behavioral practices aimed at fostering happiness contribute to the patients' acceptance of their condition, promote behavioral changes that support better self‐care, and help eliminate existing psychological barriers to effective disease management [35].

Fatigue is an undefined feeling and a subjective complaint of weariness. It is a multidimensional feeling with diverse definitions, encompassing physical (low energy and need for rest), cognitive (difficulties in maintaining focus and attention), and emotional aspects (reduced motivation or interest) [9].

The current study demonstrated that the mean perceived fatigue score before the intervention, indicating a moderate to high fatigue. This result aligns with the findings of the study conducted by Nouri Azari et al. and Bayat Asghari et al. [17, 36].

Overall, the findings indicate that fatigue levels in the control group remained largely unchanged, with only minimal fluctuations that did not reach statistical significance. This stability suggests that, in the absence of a targeted psychological intervention, perceived fatigue in patients with type II diabetes tends to persist over time. The minor variations observed may be explained by the subjective and multidimensional nature of fatigue, which can be influenced by temporary psychological or contextual factors during repeated measurements rather than by true clinical change [37].

In contrast, the present results support the notion that happiness training programs can meaningfully influence psychological constructs such as perceived fatigue and SOC through well‐established cognitive and behavioral mechanisms. From a cognitive standpoint, happiness‐based interventions emphasize positive cognitive restructuring, gratitude, and adaptive reappraisal of stressful experiences. These processes help individuals reinterpret illness‐related stressors as more manageable and less threatening, thereby reducing emotional exhaustion and perceived fatigue [38].

From a behavioral perspective, happiness training encourages greater engagement in pleasurable and meaningful activities, enhances social connectedness, and strengthens a sense of purpose and personal control. Such behavioral changes are particularly relevant for individuals with chronic conditions like type II diabetes, where continuous self‐management demands may lead to long‐term psychological strain and reduced energy levels. By fostering resilience and reinforcing coping resources, happiness‐based interventions appear to improve SOC and support higher vitality, ultimately contributing to reduced perceived fatigue [39].

Moreover, by strengthening individuals' SOC—the capacity to view life as comprehensible, manageable, and meaningful—happiness training can empower these patients to better cope with life's challenges happen due to their health condition, leading to improved psychological functioning and reduced vulnerability to emotional exhaustion [24].

Taken together, these findings highlight the potential value of integrating structured happiness training programs into comprehensive diabetes care models. Addressing psychological well‐being alongside medical management may enhance patients' adaptive capacity, improve coherence, and alleviate fatigue, thereby supporting more sustainable long‐term disease management.

### 4.1 The Study Limitations

This study has a limitation as it used convenience sampling for patient recruitment. Since participants were included according to their accessibility and willingness rather than through a probability sampling method, this might have caused selection bias and limited the generalizability of the results. Although random allocation via the lottery method was used to assign patients to intervention and control groups, the initial nonrandom sampling approach could affect the representativeness of the study population. Future studies should consider employing probabilistic sampling techniques to enhance external validity.

One limitation of the present study is that fatigue was assessed using only the total score of the MFI, and analyses of its individual subscales were not performed. As a result, potential differential effects of the happiness training program on specific dimensions of fatigue—such as general, physical, mental fatigue, reduced activity, and reduced motivation—could not be examined. Future studies are therefore recommended to analyze MFI subscales separately to provide a more nuanced and comprehensive understanding of how happiness‐based interventions influence distinct aspects of fatigue. One limitation of this study is the increased risk of Type I error due to multiple *t*‐tests, which may lead to false‐positive findings despite attempts to control for this risk.

## 5 Conclusion

Based on the results, it can be inferred that happiness training contributes to enhancing the SOC and reducing perceived fatigue to enhance and cultivate a healthy relationship with the self, healthy interpersonal relationship in life, and self‐confidence.

By fostering positive thinking, happiness training culminates in having a joyful life, might lead to reducing psychological tensions, and building self‐confidence, all of which contribute to improving the mental health status for patients with type II diabetes.

The results can be applied to the planning improvement in the mental health of diabetic patients. Given the broad and emerging concepts of SOC and perceived fatigue, extensive and multifaceted studies is required in this field to achieve better outcomes in the control and treatment of chronic diseases, including type II diabetes, while taking into account inter and intraindividual differences.

## Author Contributions

Mahdieh Poodineh Moghadam conducted the initial design of the research and collected data. Mahdieh Poodineh Moghadam, Mohammad Hassan Ahmadi Sartakhti, and Najmeh Ahmadpour conceptualized the study, analyzed and interpreted the data. Moreover, Mahdieh Poodineh Moghadam, Mohammad Hassan Ahmadi Sartakhti, and Najmeh Ahmadpour prepared the draft and revised the study. Mahdieh Poodineh Moghadam, Mohammad Hassan Ahmadi Sartakhti, Najmeh Ahmadpour, and Tayebe Sargazishad read and approved the final manuscript and had full access to all the data of the research and take complete responsibility for the integrity of the data and the accuracy of the data analysis. The [corresponding author] confirms that this manuscript is an honest, accurate, and comprehensive account of the reported research; that no significant aspect of the study has been removed; and that any discrepancies from the study as planned (and, if relevant, registered) have been clearly addressed.

## Funding

The authors have nothing to report.

## Consent

All authors have approved the manuscript and consent to its publication in this journal.

## Conflicts of Interest

The authors declare no conflicts of interest.

## Transparency Statement

The lead author Mahdieh Poodineh Moghadam affirms that this manuscript is an honest, accurate, and transparent account of the study being reported; that no important aspects of the study have been omitted; and that any discrepancies from the study as planned (and, if relevant, registered) have been explained.

## Data Availability

Data supporting the results of the present research can be collected from the corresponding author and first author upon reasonable request.
